# Patterns of Cell Division, Cell Differentiation and Cell Elongation in Epidermis and Cortex of *Arabidopsis* pedicels in the Wild Type and in *erecta*

**DOI:** 10.1371/journal.pone.0046262

**Published:** 2012-09-25

**Authors:** Mark G. R. Bundy, Olivia A. Thompson, Matthew T. Sieger, Elena D. Shpak

**Affiliations:** Department of Biochemistry, Cellular and Molecular Biology, University of Tennessee, Knoxville, Tennessee, United States of America; University of Nottingham, United Kingdom

## Abstract

Plant organ shape and size are established during growth by a predictable, controlled sequence of cell proliferation, differentiation, and elongation. To understand the regulation and coordination of these processes, we studied the temporal behavior of epidermal and cortex cells in *Arabidopsis* pedicels and used computational modeling to analyze cell behavior in tissues. Pedicels offer multiple advantages for such a study, as their growth is determinate, mostly one dimensional, and epidermis differentiation is uniform along the proximodistal axis. Three developmental stages were distinguished during pedicel growth: a proliferative stage, a stomata differentiation stage, and a cell elongation stage. Throughout the first two stages pedicel growth is exponential, while during the final stage growth becomes linear and depends on flower fertilization. During the first stage, the average cell cycle duration in the cortex and during symmetric divisions of epidermal cells was constant and cells divided at a fairly specific size. We also examined the mutant of *ERECTA*, a gene with strong influence on pedicel growth. We demonstrate that during the first two stages of pedicel development *ERECTA* is important for the rate of cell growth along the proximodistal axis and for cell cycle duration in epidermis and cortex. The second function of *ERECTA* is to prolong the proliferative phase and inhibit premature cell differentiation in the epidermis. Comparison of epidermis development in the wild type and *erecta* suggests that differentiation is a synchronized event in which the stomata differentiation and the transition of pavement cells from proliferation to expansion are intimately connected.

## Introduction

From a seed no bigger than that of a cucumber, California's coastal redwood tree can grow to a height of more than 350 feet. At the same time, aquatic watermeal plants are so small that they resemble specks of cornmeal. The nature of the mechanisms controlling the size and shape of organs is an important but still unanswered question of developmental biology. Two factors determine the size of mature organs: cell number and cell size. A current model for metazoans proposes that cell number and cell size are controlled by distinct proliferation and growth signals that negatively affect each other [1]; when cellular size is increased, cell count is reduced and vice versa. This regulation ensures that induced alterations in cell proliferation are compensated for by changes in cell size, resulting in little net change in the final organ size. The compensation phenomenon has also been observed in plants [2]–[6]; however, it is not clear whether the model proposed for metazoans can be directly applied to plants [7]; [8]. First of all, the majority of cell elongation occurs after termination of cell division and, therefore, the actions of proliferation factors and cell expansion factors are separated in time. In addition, some signals, such as auxin, regulate both proliferation and elongation of cells [9]–[11].

To understand regulation of organ size and the molecular mechanism of compensation it is essential to know the signaling pathways that control and coordinate cell proliferation and cell elongation. Plant hormones are the most obvious candidates for that role, with cytokinins being implicated in regulation of cell proliferation [12]–[14]; and gibberellins, brassinosteroids, and auxin regulating both cell proliferation and cell elongation [9]–[11], [15]–[19]. Despite these advances, a detailed understanding of the role of hormones in the coordination of cell behavior in tissues and organs is still elusive, with such central questions as the spatial-temporal pattern of hormone action and the identity of the downstream targets remaining unanswered. Moreover, other signaling pathways initiated at the plasma membrane by receptor-like kinases also play a role in the coordination of cell behavior during tissue growth [20].

Here, we explore the role of the receptor-like kinase ERECTA (ER) in regulation of organ growth. In *Arabidopsis* there are two paralogs of ER; ERL1 and ERL2 (for ERECTA-LIKE 1 and 2). This family of genes is involved in the controls of stomatal patterning. In the early stages of epidermal development, all protodermal cells undergo symmetric proliferative divisions. Differentiation of a stoma is initiated by asymmetric division of a select group of protodermal cells called meristemoid mother cells (MMCs). This asymmetric division produces a small triangular meristemoid cell that can undergo several additional rounds of asymmetric division, but will eventually differentiate into a round guard mother cell that divides symmetrically to generate a pair of guard cells. Analysis of different combinations of mutants determined that ER, ERL1, and ERL2 inhibit the initial decision of protodermal cells to become a MMC [21]. In addition, ERL1 and, to a lesser extent, ERL2 are important for maintaining the potential of meristemoid cells to divide asymmetrically [21]. Many components of this signaling pathway have been identified based on their involvement in regulation of stomatal development (for a review see [22]).

In contrast, the function of the *ER* gene family in the regulation of plant size is less clear. The single *er* mutation reduces the size of *Arabidopsis* plants. It confers a more compact inflorescence with flowers clustered on the top, as a result of reduced elongation of internodes and pedicels [23]. The decreased length of *er* pedicels correlates with a reduced number of cortex cells, which are on average slightly bigger than in the wild type [24], [25]. Additional mutations in the *ER* gene family further reduce the cortex cell count, with enlarged, irregularly shaped cells and multiple gaps between cells [26]. The loss of *ER* function not only diminishes cortex cell proliferation but also causes reduced pavement cell expansion and increased duration of asymmetric cell divisions in leaves [27]. Recently, the regulation of organ elongation by the *ER* family genes was attributed to their function in the phloem where they perceive a signal from the endodermis [28]. This finding suggests that interlayer communication between the endodermis and phloem plays an important role in determining the stem length. The function of the *ER* family genes in the development and growth of phloem and the impact on other tissues is not clear. To gain a better understanding of the consequences of the *er* mutation on the cortex and epidermis, and how it contributes to the whole organ growth over time, we investigated the phenotype of the null *er* mutant.

One of our major goals was to analyze the *er* phenotype at different times, from the moment of organ initiation to maturity, and in different tissue layers. Roots and leaves are the model plant organs to study kinetics of growth [29]–[32]. The *ER* family genes function only in aboveground plant organs, which makes roots an inappropriate system. Leaf size is only marginally affected in the single *er* mutant [27], and while leaf size is dramatically reduced in the *er erl1 erl2* mutant, the alteration of leaf initiation rate (our unpublished data) prevents temporal analysis of internal leaf tissues on fixed samples. Fortunately, the mutation in *ER* strongly affects pedicel growth (Figure S1; and [25]). A pedicel is a stem-like organ that attaches single flowers to the main stem of the inflorescence. It is a convenient target of study as growth occurs largely in one dimension and is determinate, there are no trichomes in the epidermis, and pavement cells have no lobes. In addition, the age of very young pedicels can be calibrated by observing the developmental stage of flowers.

Here we report the dynamics of cell proliferation, cell differentiation, and cell elongation in the epidermis and cortex during pedicel growth in the wild type and the *er* mutant. Based on examination of wild type pedicels we propose that there are three stages of pedicel development: a proliferative stage, a stomata differentiation stage and a cell elongation stage. Our analysis uncovered coordination of cell behavior within tissues and between different tissues: the onset of stomata differentiation was linked to pavement cell elongation, the termination of asymmetric cell divisions in the epidermis was followed by acceleration of the cell cycle in the cortex, and the termination of stomata differentiation was coincidental with cortex cell elongation. We observed that during the final stage of development pedicel growth was dependent on flower fertilization, and we propose that some unknown signal coming from the flower promotes cell elongation in the pedicel. Detailed temporal analysis of *er* revealed that the mutation affects the growth rate during the first two stages of pedicel development. In the cortex and epidermis of the mutant we observed a decreased cell growth rate and increased cell cycle duration but only very subtle changes in the size of cells at division. In *er* epidermis meristemoid differentiation was premature and prolonged. Interestingly, the prolonged period of asymmetric divisions in the epidermis of the mutant was coincidental with a lack of cell cycle acceleration in the cortex. Our investigation demonstrates that pedicels are a useful model for studying the coordination and interdependence of different tissues during plant organ development.

## Materials and Methods

### Plant materials and growth conditions

The *Arabidopsis* ecotype Columbia (Col) was used as a wild type. The *er-105* mutant used in this study has been previously described [23]. Plants were grown in a soil mixture (Pro-mix PGX (Premier Horticulture Inc): Vermiculite (Palmetto Vermiculite Co.) 1∶1) supplemented with Miracle-Gro (Scotts) and approximately 3.5 mg/cm^3^ Osmocoat 15-9-12 (Scotts) under 100 lmol/m^2^/s light intensity (cool light fluorescent lighting) with an 18-h-light/6-h-dark cycle at 21°C.

### DIC microscopy

Samples were fixed overnight with ethanol: acetic acid (9∶1), rehydrated with ethanol series to 70% ethanol, cleared in chloral hydrate solution (chloral hydrate: H_2_O: glycerol 8∶1∶1) overnight and observed using Differential Interference Contrast (DIC) optics. Measurements of pedicel and cell length were performed using the NIS Elements BR program (Nikon). Cell length was measured in the middle of a pedicel at the abaxial side unless noted otherwise. Individual cell length measurements had a resolution of ±1 µm. The goal was to measure 110–125 cells per pedicel; however, in several very short pedicels a smaller number of cells were measured (but no less than 35) due to the small number of cells on the abaxial side in the middle of such pedicels. During analysis of epidermal cells the lengths of meristemoids, guard mother cells and stomata were not measured. Cortex cells were measured only in the cell layer directly adjacent to the epidermis. For analysis of cell size changes along the proximodistal axis and on the abaxial versus adaxial sides, we measured the length of 50–60 cells. The identity of meristemoids and GMCs was established based on their shape; triangular for meristemoids and oval for GMCs (Figure S2).

### *In vivo* measurements of pedicel growth

Pedicel length was measured each day, in the middle of the day, starting from ∼1 mm long until no increase in growth was observed. For accuracy, we measured each pedicel with calipers four times and the average was used as the measurement value. The 6^th^ to 11^th^ pedicels from the bottom on the main inflorescence stem were selected for measurements. No more than 3 pedicels were measured on a single inflorescence stem. In each experiment, the growth of multiple pedicels was monitored at every data point and the average was determined.

### Monte Carlo simulation of cell size distributions

All simulations of cell length distributions began with 3 initial cells based on estimation of the initial pedicel length of ∼32 µm from growth curve analysis and the average cell size of epidermal and cortex cells between 11.5 and 13 µm. Cell starting lengths were randomly sampled from the normal distribution
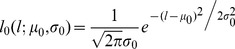
(1)with 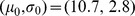, values chosen to give the same mean pedicel size at *t* = 0 as the growth curve analysis and standard deviation as early cortex data. Division lengths *l_d_* were randomly sampled from a separate normal distribution 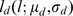. Cells then grew independently of all other cells, with discrete time steps 1 min in duration. Halving the time step duration did not significantly alter the results. With each time step, the length of a cell increased by the amount 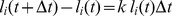, where *k* was the rate of cell growth (units h^−1^), 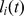 was the length of the *i*th cell at time *t*, and 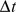 was the time step. If the new length of the *i*th cell exceeded the cell division length *l_d,i_* previously sampled for the cell, it then divided symmetrically into two daughter cells. Each daughter cell was assigned a new randomly sampled division length, and the procedure was repeated until the total duration of the simulation had elapsed. Total pedicel length was calculated by summing the individual lengths of cells in a simulated pedicel.

**Figure 1:**
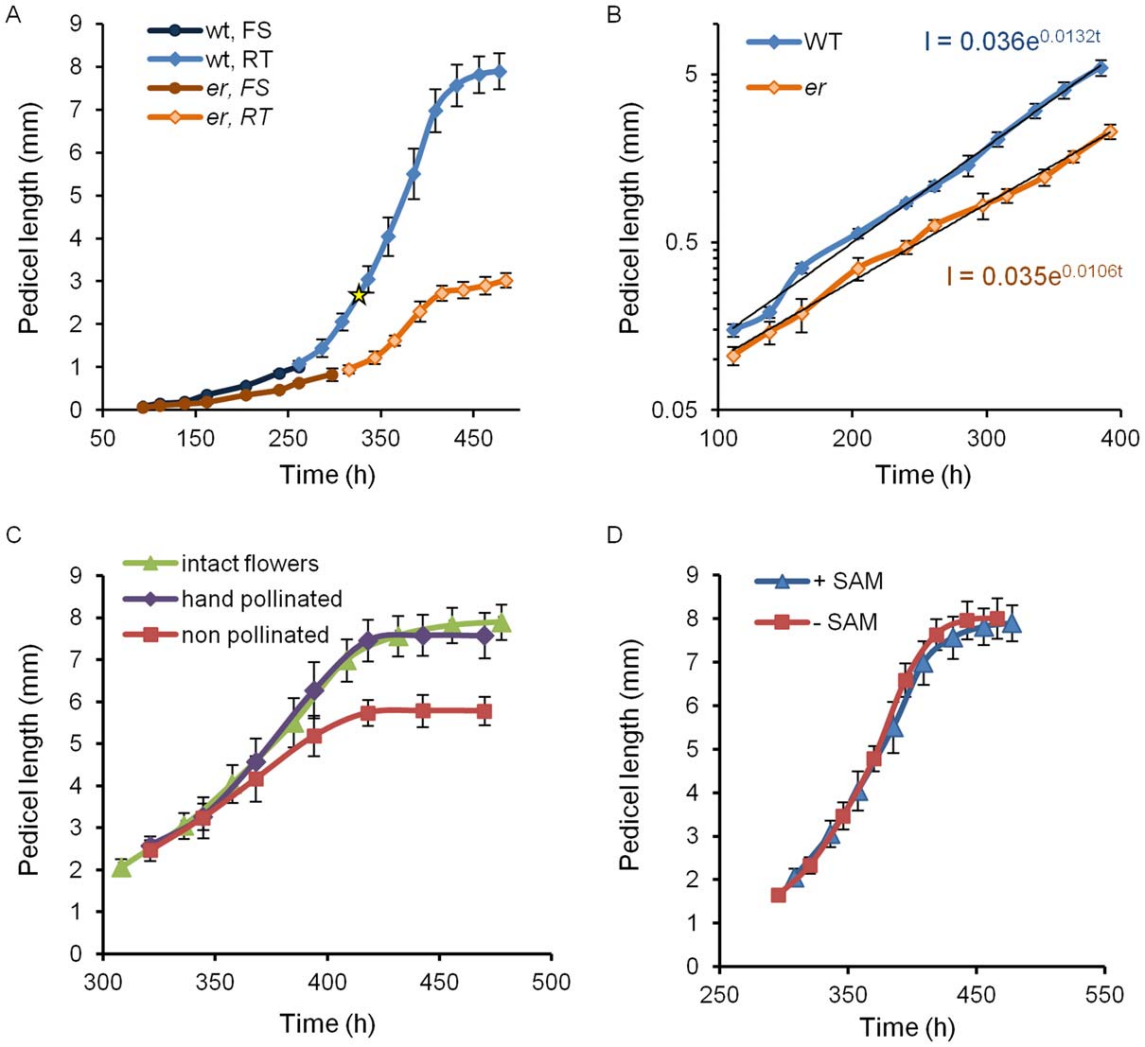
The analysis of *Arabidopsis* pedicel growth over time. A. Comparison of wild type and *er* pedicel length over time demonstrates that while growth occurs for the same total time, *er* pedicels grow more slowly. During early pedicel development the length was determined by DIC microscopy of fixed samples (FS) and the age was decided based on the flower stage; n = 5–12. Starting from 1 mm for wt and 0.95 mm for *er* the length was measured in real time (RT) and averaged for 9 samples. The yellow star represents the time when fertilization occurred in the wild type. B. Pedicel elongation is exponential for the first 16 days. The best fit exponential growth curve is shown: l = 0.036e^0.0132t^ for wt and l = 0.035e^0.0106t^ for *er*. The y axis is a logarithmic scale. C. Pedicel growth depends on fertilization of flower; n = 6–7. D. Pedicel growth after 300 hours does not depend on the presence of SAM; n = 7–8. A–D Error bars were added to all data points and are ± SD. A–D Time zero is when a flower buttress arises on a meristem.

At the crossing of each time period boundary, the cell division length distribution parameters 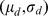 were allowed to change and a new division length was resampled for all existing cells. Within a given time period 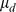 and 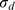 remained constant. All other simulation parameters were fixed across all time periods. The cell growth rate factor *k* was determined from the fits to experimental data shown in Figures 1B; *k* = 0.0132 for the wild type and *k* = 0.0106 for *erecta*. Cortex and epidermis cells were modeled separately, with no interaction between them.

The simulation was repeated for 1000 individual pedicels, and the results averaged. Cell size histograms were generated in the same manner as the experimental data; cell sizes “measured” in a specified time period were binned, and the fraction of total cells in each bin was computed. No scaling factors were used. The results were then compared to the data. The random sampling used in the simulation produces results that are unique for each simulation run; however, the run-to-run variation of the fraction of cells in a size bin was on the order of ±0.002, much smaller than the experimental error bars.

The best-fit cell division length distribution parameters 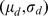 for each time period were determined by the method of least-squares, in which the sum of squares was minimized:
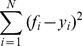
(2)where *y_i_* was the fraction of cells in the *i*th bin of experimental data, *f_i_* was the same for simulated data, and *N* was the number of bins.

## Results

### Elongation of pedicels in *Arabidopsis* is exponential for the first 16 days, and is increased by fertilization of ovules

Two approaches were used to measure the rate of pedicel elongation. Since flowers in *Arabidopsis* develop in 20 distinct visually identifiable stages [33] concurrent with the pedicel growth, we exploited these stages (specifically stages 5 to 11) to calibrate growth during early development and measured pedicel length on fixed samples using DIC microscopy. After stage 11, pedicels were long enough (>1 mm) to measure the rate of growth directly on live plants.

At stage 5 a pedicel is connected to a four-day-old bud that has all flower organ primordia formed and bulging, but it is not yet enclosed in sepals [33]. Wild type pedicels at this stage were 0.08±0.01 mm long (values are averages and uncertainties are ±1 standard error unless otherwise indicated) and did not yet have a distal bulge, which develops later in mid stage nine [34]. We adopted the durations of stages 5 to 11 from Smyth et. al., 1990 [33], where they were estimated to the nearest 6 hours. Based upon this reference, we assumed that about 96 hours elapsed from formation of a flower buttress to the middle of stage 5, and about 168 hours from the middle of stage 5 to the middle of stage 11. We determined experimentally the time required for a pedicel to grow from the middle of stage 11 (pedicel length = 1.08±0.02 mm) to full length (pedicel length = 7.90±0.14 mm) to be 216 hours on average (Fig. 1A). Based on these measurements, a pedicel grows for a total of approximately 480 hours (or 20 days) from the moment a flower buttress arises on an inflorescence meristem to full length.

Our analysis of the pedicel growth curve (total length vs. time) suggested that growth was exponential for the first 16 days after which it slowed. The pedicel length vs. time data were fitted to a simple exponential growth curve 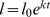, where *l* is the pedicel length (mm), *t* is time (h), *l*_0_ is the initial length (mm) of the pedicel at time zero, and *k* is the growth constant (h^−1^). The best fit to our data was obtained with the parameter values *l*_0_ = 0.0358±0.0026 mm and *k* = 0.0132±0.0003 (Fig. 1B). The average pedicel doubling time was calculated from the growth constant to be ln(2)/0.0132 = 52.5 *h*. We were able to reproduce the same curve in multiple experiments, provided that the plants were not excessively stressed by repeated handling during measurements. Stress to the plants led to a significant decrease in the growth rate and had a minor effect on the duration of growth (Figure S3). The exponential nature of pedicel growth up to 400 h has been reported previously [35] although their parameters of growth are different since a different ecotype (Lansberg erecta) was used for analysis.

It has been shown previously that silique growth strongly depends on flower fertilization [36]. We investigated whether flower fertilization has an effect on the growth of pedicels. Pedicel growth was analyzed in three situations: when the flower was undisturbed; when sepals, petals and stamens were removed and then the pistil was hand pollinated; and when the above mentioned flower organs were removed and the pistil was not pollinated. Removal of flower organs did not affect pedicel growth (Fig. 1C). Fertilization was important for the rate of growth but not its duration (Fig. 1C). After fertilization, growth continued for 4 days in both cases, but pedicels carrying unfertilized flowers grew more slowly and were shorter (5.78±0.14 mm unfertilized versus 7.57±0.21 mm fertilized). Fertilization triggers auxin and gibberellin biosynthesis in siliques [37], [38] and the flow of these hormones through the pedicel might be necessary to maintain its high growth rate. Since pedicels develop in close proximity to the inflorescence meristem, we investigated whether the meristem affects their growth by removing the meristem and monitoring the growth of a pedicel attached to the flower at stage 12 (shortly before fertilization). The removal of the meristem at that stage did not change the rate of pedicel growth (Fig. 1D).

An organ grows due to growth and division of its cells with both of these processes being coordinated at the tissue levels and between different layers. As in many other plant organs, there are three tissues in pedicels: epidermis, cortex/mesophyll, and vasculature. Here we describe the behavior of cells in the epidermis and cortex and ignore for now the vasculature. We use the term ‘cell proliferation’ to refer to cells that grow and then divide, and the term ‘cell expansion’ to refer to cell growth that is not associated with cell divisions.

### Patterns of division and elongation of epidermal cells during pedicel growth

To characterize epidermis development we measured cell length and analyzed the formation of stomata in fixed pedicels from 0.120 mm long (late stage 5) to mature size. Cells were measured in the middle of the pedicel on the abaxial side unless otherwise stated. Up to 5.5 mm of length (∼390 h), pedicel age was calculated using the fitted exponential growth curve (Fig. 1B) by substituting *l* with measured pedicel length and solving for *t*. From 5.5 mm until 7 mm we used the equation *l* = 0.063 *t* -18.76, where *l* is the pedicel length (mm), and *t* is time (h) to describe linear growth. Our analysis of cell behavior in the epidermis identified 3 major developmental stages (Table 1):

**Table 1:** Characteristics of the three stages of pedicel growth in the wild type.

	Alternative name	Growth (best fit)	Duration	Final pedicel length	Major events in epidermis	Major events in cortex
Stage I	Proliferative stage	Exponential	∼240 h	∼0.85 mm	Symmetric and asymmetric cell divisions	Symmetric cell divisions
Stage II	Stomata differentiation stage	Exponential	∼100 h	∼3.1 mm	Asymmetric cell divisions, differentiation of stomata, cell elongation	Symmetric cell divisions
Stage III	Cell elongation stage	Exponential/linear	∼110 h	∼7.6 mm	Cell elongation	Cell elongation, few cell divisions

#### Stage I or “Proliferative stage”

**Figure 2:**
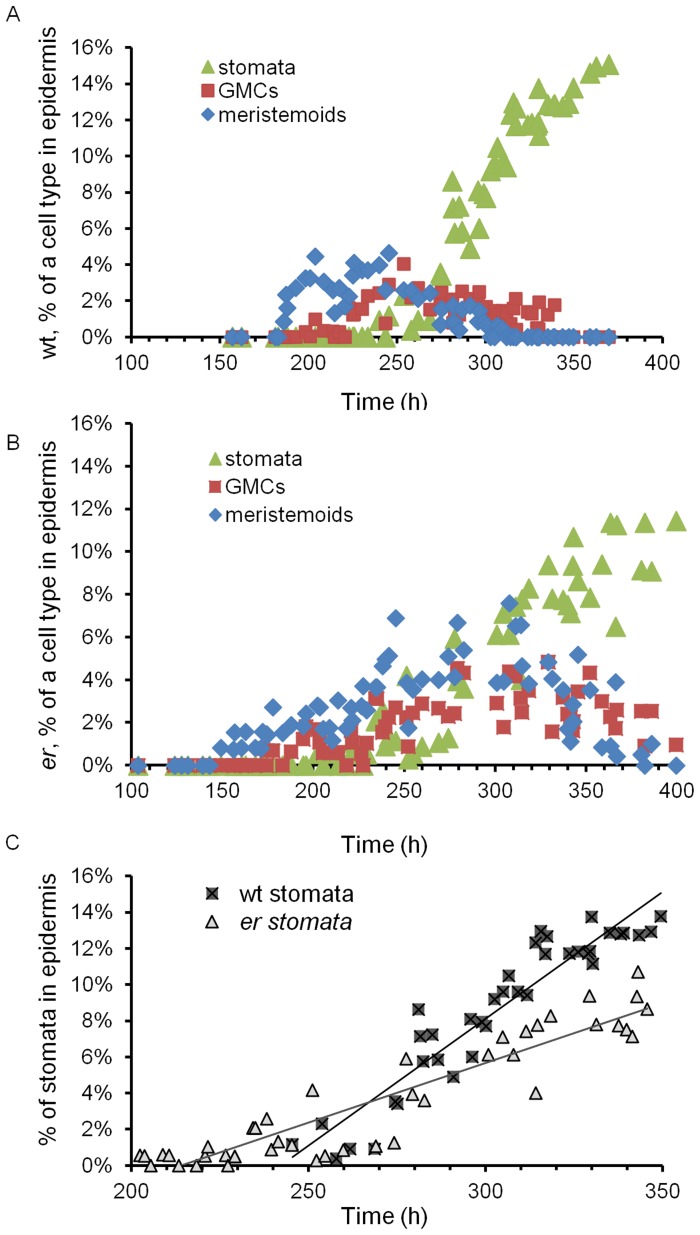
Formation of meristemoids, guard mother cells, and stomata in the wild type and er pedicels. Formation of meristemoids, guard mother cells and stomata in the wild type (A) and in *er* (B) over time. Comparison of stomata formation in the wild type and *er* suggests an early onset of differentiation but slower rate in *er* (C). Data points represent the fraction of a cell type in an individual pedicel. The pedicel age (h) was determined based on pedicel length as described in the text. The decrease in the number of meristemoids in *er* after 350 h (B) is due to cell elongation and not cell differentiation into GMCs.

**Figure 3:**
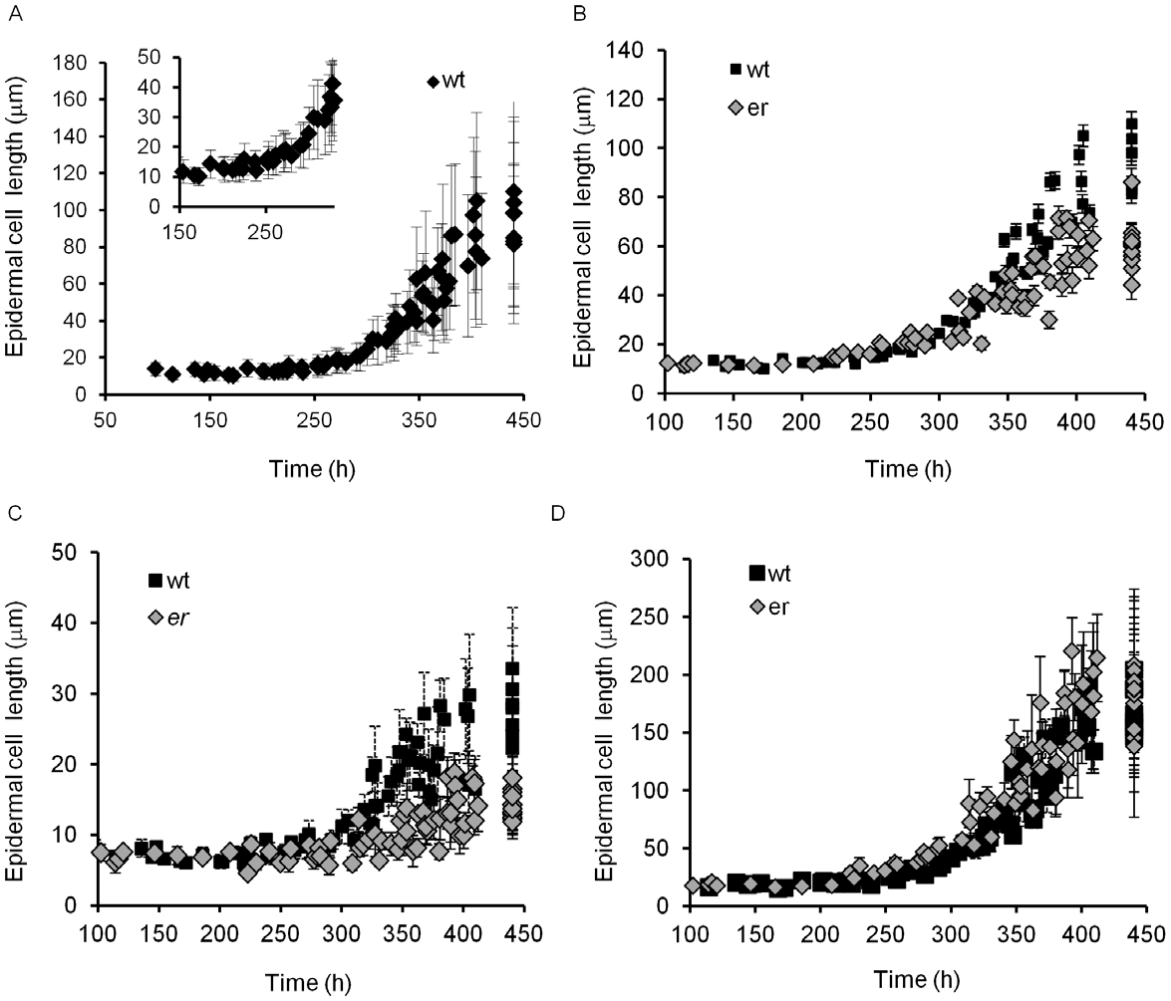
Changes of epidermal cell length in *er* pedicels versus the wild type over time. A. The average size of epidermal cells in the wild type during pedicel growth. The insert demonstrates an increase of cell size at 250 h. B–D. Comparisons of epidermal cell sizes in *er* and the wild type during pedicel growth: the average cell size (B); the average size of 10% of the shortest cells (C); the average size of 10% of the longest cells (D). A–D. Every data point represents an average cell size in an individual pedicel. N = 35–125. Error bars are ± SD in A, C, D and SE in B. The pedicel age (h) was determined based on pedicel length as described in the text. The length of meristemoids, guard mother cells and stomata is excluded from the presented data.

**Figure 4:**
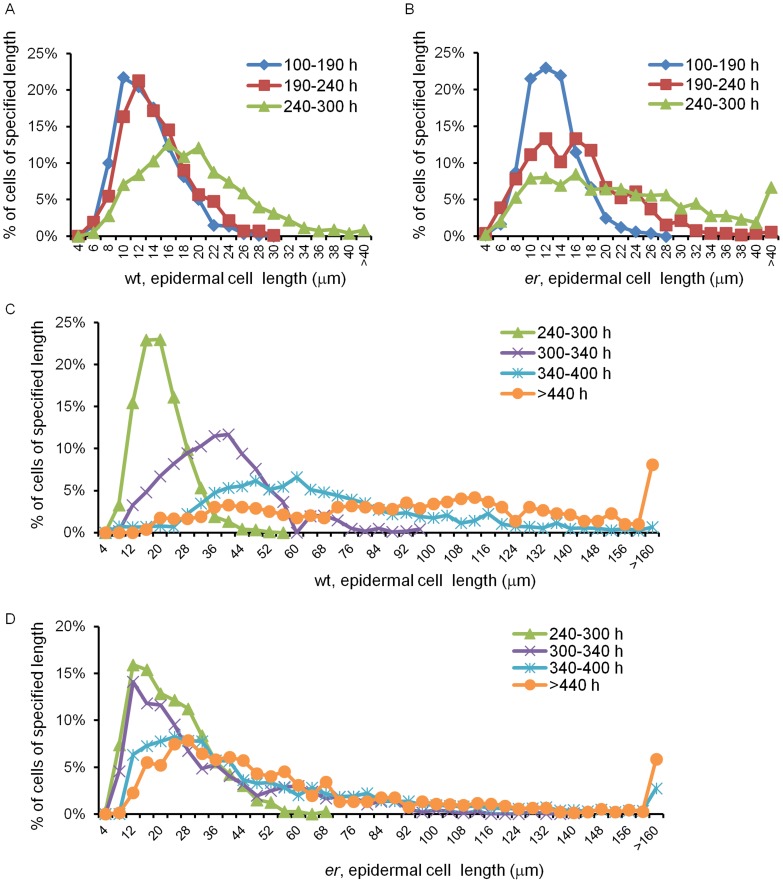
Analysis of epidermal cell size distribution in pedicels of different ages. Data points represent the percentage of cells of a specified length with bins at 4 µm representing cells that are 4 µm or smaller, bin at 6 µm representing cells 4 to 6 µm long, etc. Bin widths for A and B are 2 mm. Bin widths for C and D are 4 µm. Data series represent pedicels of different ages and are color coded. Age of pedicels is given in hours (e.g. 100–190 h). The wild type cell size distribution is in A and C and *er* cell size distribution is in B and D. Number of cells in distributions 100–190 h and 190–240 h are between 479 and 983; 240–300 h and 300–340 h are between 964 and 1224; 340–400 h 2043 and 2681; >440 h 790 and 1364. The length of meristemoids, guard mother cells and stomata is excluded from the presented data.

**Figure 5:**
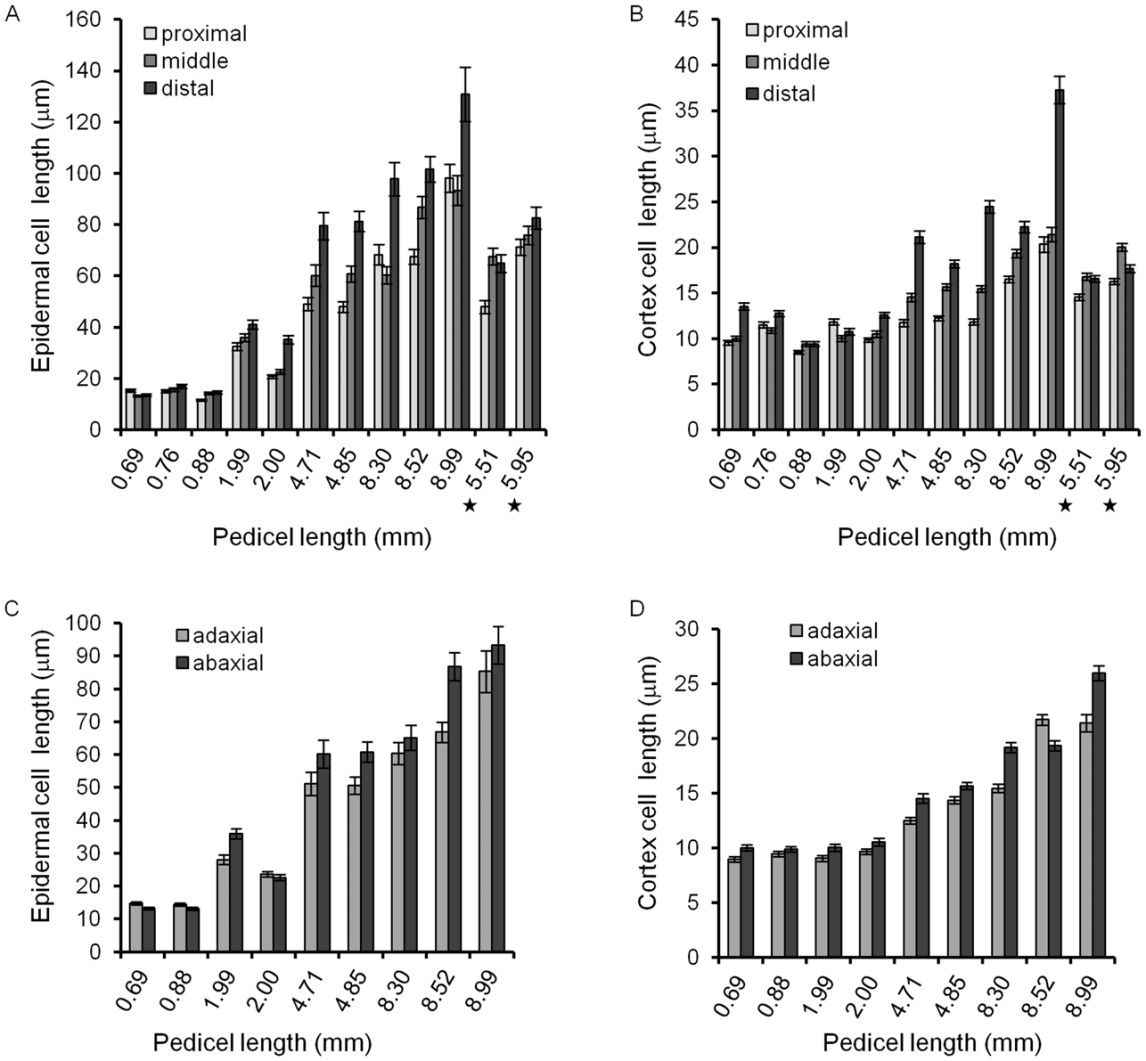
Average cell length along proximodistal axis and on abaxial and adaxial sides in wt pedicels. In older pedicels (>4 mm) elongation of epidermal (A) and cortex (B) cells at the distal end of the pedicel significantly exceeds elongation at the proximal end. The difference in cell elongation along the proximodistal axis in pedicels attached to unfertilized flowers (labeled with a star) is dramatically reduced. Epidermal (C) and cortex (D) cells typically are slightly longer on the abaxial side of pedicels compared to the adaxial side. This difference in length is independent of pedicel age. A–D. n = 50–60 Error bars are ± SE.

This stage lasted for 240 h, from pedicel formation until it reached 0.85 mm, and consisted of two sub-stages. In pedicels shorter than ∼0.4 mm (∼185 h) all epidermal cells divided symmetrically; afterwards we observed asymmetric cell division producing meristemoids (Fig. 2A). The beginning of asymmetric divisions corresponded to flower stage 9, which is coincident with another hallmark of pedicel development – the formation of a distal bulge [34]. At a pedicel length of ∼0.5 mm (∼200 h) we observed formation of the first guard mother cells, and at ∼0.85 mm (∼240 h, middle of flower stage 10) the first stomata were detected (Fig. 2A). This timing suggests that it takes roughly 15 h for a meristemoid to differentiate into a guard mother cell and roughly 40 h for a guard mother cell to differentiate into two guard cells. In addition, we noticed that for the first ∼240 h of growth, before stomata differentiation, cells in the epidermis had a consistent average length of 12.7±0.4 µm (Fig. 3A). The longest 10% of epidermal cells in Stage I measured 19.9±0.6 µm and did not change, suggesting that cells had not yet transitioned to elongation. The formation of the first stomata at ∼240–250 h (Fig. 2A) coincided with an increase in the average epidermal cell length, which was first noticed at ∼250 h. The elongation of epidermal cells after 250 h is evident in Figure 3A. This elongation is also apparent in the distribution of epidermal cell lengths (Fig. 4A). In pedicels that were 100–190 h old and 190–240 h old the epidermal cell length distributions were very similar, but in pedicels that were 240–300 h old the distribution shifted to longer cell lengths (Fig. 4A). Measurements along the pedicel suggested that in Stage I there is little difference in epidermal cell length behavior along the proximodistal axis (Fig. 5A, see pedicels that are 0.69 mm, 0.76 mm, and 0.88 mm long). We did not observe a significant difference in cell behavior between the abaxial versus adaxial sides in the middle of the pedicel in Stage I (Fig. 5C, see pedicels that are 0.69 mm and 0.88 mm long). Therefore, measurements obtained from the middle of the abaxial side of the pedicel can be safely extrapolated to the whole organ.

We designated the initiation of stomata differentiation as the end of Stage I. At the end of this stage 10–16% of epidermal cells were products of asymmetric cell division, with half of them being meristemoids or guard mother cells and other half being stomatal lineage pavement cells. Since the average length of epidermal cells did not change during this period, the average length of a cell cycle in those cells should be equal to the pedicel doubling time (∼52.5 h).

#### Stage II or “Stomata differentiation stage”

During the next one hundred hours, as the pedicel elongated from 0.85 mm to 3.1 mm (a 3.7 times increase), the epidermis grew via elongation of pavement cells and by formation of new cells through asymmetric divisions. During this stage the average length of an epidermal cell increased from 12.7±0.4 µm to 41.7±8.2 µm (3.3 times) (Fig. 3A) and the average length of the longest 10% of cells increased from 19.9±0.6 µm to 71.8±1.8 µm (3.6 times) (Fig. 3D). The close correspondence between the magnitude of pedicel growth and magnitude of elongation in the longest 10% of cells suggests that those cells switched from cell division to elongation early in Stage II. Measurement of cell length along the pedicel detected some differences in epidermal cell elongation, with cells close to the distal end being slightly longer (Fig. 5A, see pedicels that are 1.99 mm and 2.00 mm long). At this stage we also began to observe in some pedicels a difference in epidermal cell length on the abaxial and adaxial sides, with abaxial cells being slightly longer (Fig. 5C, see a pedicel that is 1.99 mm long).

By dividing the total pedicel length by the average cell length we estimated that the number of cells in a longitudinal row increased ∼1.1 times from ∼67 cells to ∼75 cells during this stage (Table S1). Since the pedicel elongates 3.7 times, this implies that elongation of cells is the major contributor to epidermis growth in this stage. All of the small cells that were observed in this stage were located next to meristemoids, guard mother cells, or stomata, suggesting that formation of new cells is largely a result of asymmetric cell divisions. Only on very rare occasions did we detect cells symmetrically dividing parallel to the proximodistal axis and increasing the number of cells radially (Figure S4).

Pavement cell elongation (Fig. 3A) and differentiation of stomata are the main hallmarks of Stage II (Fig. 2A). The termination of this stage at ∼340 h coincides with the completion of guard mother cell differentiation into stomata. In addition, the end of this stage correlates with fertilization of flowers (pedicel length at fertilization equaled 2.93±0.08 mm, n = 8).

#### Stage III or “Cell elongation stage”

During pedicel growth from 3.1 mm to ∼7.6 mm (a 2.5 times increase), the epidermis expanded solely through cell elongation. During this stage the average length of an epidermal cell in the middle of a pedicel increased from 41.7±1.8 µm to 94.3±4.2 µm (2.3 times) (Fig. 3A) and the average length of the longest 10% of cells increased from 71.8±1.8 µm to 166.5±3.0 µm (2.3 times) (Fig. 3D). While prior to this stage epidermis cells at the proximal and distal ends differed only slightly, during Stage III their growth was significantly different, with elongation of cells at the distal end being much more prominent than in the middle or at the proximal end (Fig. 5A, see pedicels that are 4.71 mm, 4.85 mm, 8.30 mm, 8.52 and 8.99 mm long). We noted previously that fertilization is associated with longer pedicels (Fig. 1C). We observed only slight differences in epidermal cell length along the proximodistal axis in mature pedicels carrying unfertilized flowers, and cells were shorter compared to pedicels with fertilized flowers (Fig. 5A, starred data points). This observation supports the hypothesis that post fertilization the flower produces a signal that promotes cell elongation in the pedicel, especially at the end directly adjacent to the flower.

### Patterns of division and elongation of cortex cells during pedicel growth

In addition to our investigation of the epidermis, we also investigated the behavior of cortex cells in the context of the 3 stages of epidermal development (Table 1):

#### Stage I and Stage II

**Figure 6:**
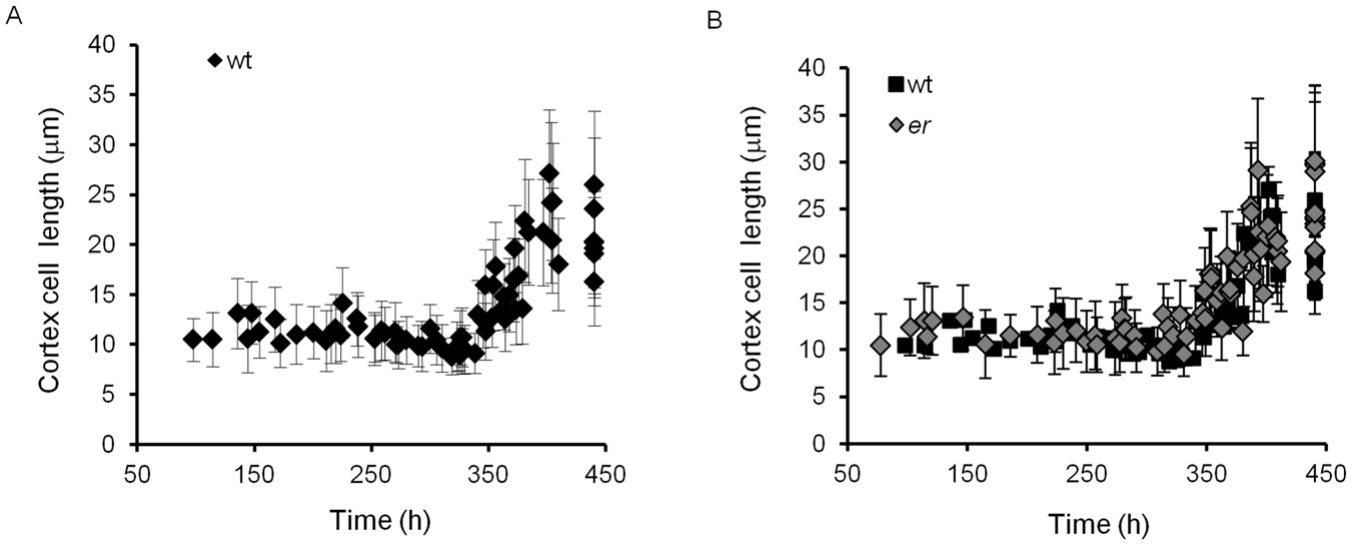
Changes of cortex cell length in pedicels over time. A. The average size of cortex cells in the wild type. B. The average size of 10% of the shortest and 10% of the longest cortex cells in the wild type. C. The average size of cortex cells in *er* compared to the wild type. D. The average size of 10% of the shortest and 10% of the longest cortex cells in *er* over time. A–D. Every data point represents an average cell size in an individual pedicel. N = 35–125. Error bars are added to all data points except the wild type on C and they are ± SD. The pedicel age (h) was determined based on pedicel length as described in the text.

**Figure 7:**
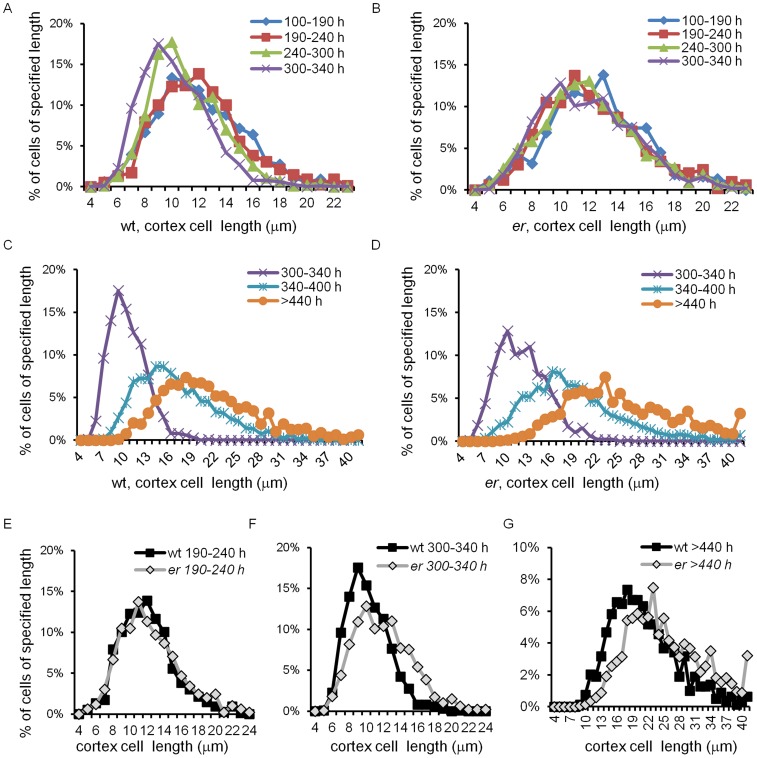
Cortex cell size distribution in pedicels of different ages in the wild type and *er*. Data points represent percentage of cells of specified length range, e.g. data point at 6 µm represents the percentage of cells 5 to 6 µm long. All bins are equal of 1 µm. The last bin on C, D and G represent cells >40 µm long. Data series represent pedicels of different ages and are color coded. Age of pedicels is given in hours (e.g. 100–190 h). The wild type cell size distribution is given in A and C and the *er* cell size distribution in B and D. Cortex cells from the wild type and *er* pedicels of the same age are compared in E, F and G. Number of cells in distributions: 100–190 h and 190–240 h between 327 and 757; 240–300 h and 300–340 h between 929 and 1020; 340–400 h 2098and 2856; >440 h 790 and 1364.

During stages one and two (0 to 240 h and 240 to 340 h, respectively) the cortex grew solely by cell proliferation. While the pedicel grew from 0.12 mm to 3.1 mm, a cursory look suggested that the average cortex cell length remained around 10.8 µm (Fig. 6A). However, the cell length distribution was different during these stages (Fig. 7A). The combined cortex cell length histogram for 100–190 hour-old pedicels was the same as for 190–240 hour-old pedicels, with average cortex cell lengths of 11.4±0.4 µm and 11.7±0.4, respectively (Table S2). Since the average length of cortex cells did not change during Stage I, the average length of a cell cycle in those cells should be equal to the pedicel doubling time (∼52.5 h). During Stage II the average length of cortex cells decreased slightly: in pedicels that are 240–300 h old the average cell length was 10.6±0.2 µm, and in pedicels that are 300–340 h old the average cell length further decreased to 9.7±0.2 µm (Table S2). The decrease of cell length was especially noticeable when examining the average length of the longest 10% of cells (Fig. S2A). Our analysis of average cell length along the proximodistal axis during Stages I and II did not reveal significant differences in cell behavior (Fig. 5B, see pedicels that are 0.69 mm, 0.76 mm, 0.88 mm, 1.99 and 2.00 mm long). Comparison of cells on the abaxial versus adaxial sides detected only a very slight increase in cell length on the abaxial side (Fig. 5D, see pedicels that are 0.69 mm, 0.88 mm, 1.99 and 2.00 mm long).

In summary, during Stage I the average cell cycle duration in cortex cells does not change (∼52.5 h) and is equal to the cell cycle duration in the epidermis. During Stage II cortex cells divide at a smaller size and the rate of cell cycle increases. Interestingly, this decrease in the duration of cell cycle is correlated with a change in epidermal cell behavior where few cells are dividing and most are elongating.

#### Stage III

At approximately 340 hours, when differentiation of stomata in the epidermis was completed, cell proliferation in the cortex slowed significantly and cells began to elongate (Fig. 6A, Fig. 7C). From 340 hours to maturity pedicels elongated ∼2.5 times and the average cortex cell length in the middle of the pedicel increased 2.1 times, from 9.7±0.2 µm to 20.7±1.2 µm (Fig. 6A). All cells in the population elongated, with the smallest 10% of cells and the longest 10% of cells elongating ∼1.9 times (from 6.4±0.1 µm to 12.5±0.5 µm) and ∼2.2 times (14.1±0.5 µm to 31.3±1.9 µm), respectively (Fig. S2). The cell length distribution broadened considerably compared to the previous stages (Fig. 7C), which can be explained by differential cell elongation and/or by exponential cell growth; i.e., the cell elongation rate increasing as cell size increases [39]. Since the distribution broadens and not narrows, it is unlikely that small cells grow faster than bigger cells, as was observed in epidermal cells of leaves [31]. Just as in the epidermis, during this stage we observed differential elongation of cortex cells along the proximodistal axis, with greater elongation at the distal end (Fig. 5B, see pedicels that are 4.71 mm, 4.85 mm, 8.30 mm, 8.52 and 8.99 mm long). This difference in cell elongation along the pedicel length was absent in pedicels attached to unfertilized flowers (Fig. 5B starred data points).

### The effect of the *er* mutation on pedicel growth

Mutation in the *ER* gene noticeably reduces plant size. Both plant height and pedicel length are reduced by approximately a factor of two in the mutant [23]. Analysis of mature *er* pedicels revealed two defects: a reduced number of cortex cells [25] and reduced elongation of pavement cells in the epidermis [21]. To gain deeper insight into the role of ER as a regulator of plant organ size and the effect of the *er* mutation on the epidermis and cortex over time, we analyzed the growth and development of *er* pedicels.

To determine the pedicel growth curve we used the same two approaches as for the wild type: measurement of young pedicel length on fixed samples and measurement of pedicel growth *in vivo* at later developmental stages (beginning at the late flower stage 12, when the average pedicel length was 0.95 mm). As expected, we observed severely reduced elongation of pedicels in *er*, with *er* pedicels being on average 2.6 times shorter than the wild type at maturity (Fig. 1A). However, in *er*, just as in the wild type, 90% of pedicel growth occurred in the first 16 days followed by an additional four days of slight elongation, indicating that the mutation did not affect the total duration of growth. Pedicel growth in *er* was exponential for the first 16 days (Fig. 1B). The growth of pedicels was again fitted to a simple exponential growth curve 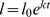. The best fit to *er* data was obtained with the parameter values *l*_0_ = 0.0350±0.0020 mm and *k* = 0.0106±0.0002 h^−1^ (Fig. 1B). This data allowed us to calculate the average pedicel doubling time in the first 16 days: ln(2)/0.0107 = 65.4 *h*, which was ∼12.9 hours longer than the wild type. The difference in pedicel length was apparent from the beginning of our measurements, at flower stage five. At stage five, *er* pedicels were 60±1 µm long versus 82±8 µm long in the wild type, and at stage six *er* pedicels were 106±6 µm versus 150±13 µm long in the wild type. In both cases the difference in pedicel length was statistically significant (p<0.05 based on Student's *t*-test). This result is consistent with a previous observation that *er* pedicels are already shorter by stage 3 of flower development [34]. Therefore, we conclude that the *er* mutation results in a reduced rate of growth over a large fraction of the growth period, but it does not change the total duration of growth.

### The *ER* gene promotes cell proliferation in the cortex and epidermis and inhibits the onset of stomata differentiation

Next we analyzed the effect of the *er* mutation on growth in the epidermis and cortex in the context of the growth stages identified in the wild type.

#### Stage I

By the end of Stage I (∼240 h), *er* pedicels were on average 0.47±0.02 mm long or ∼1.8 times shorter than the wild type (Fig. 1A). The decrease in organ growth rate suggests slower elongation of constituent cells. At this time, the length of epidermal and cortex cells in the mutant was very similar to the wild type (Fig. 3B and 6B); the average length of epidermal cells in *er* was 12.9±0.6 µm versus 12.7±0.4 µm in the wild type and the average length of cortex cells in *er* was 12.0±0.3 µm versus 11.5±0.3 µm in the wild type implying that cell division is still connected to a rather specific cell size. Since the average length of epidermal and cortex cells did not change during Stage I, the average duration of a cell cycle in those cells is equal to the pedicel doubling time (∼65.4 h), which is 12.9 h longer than in the wild type. The cortex cell length distribution did not exhibit any significant differences from the wild type (Fig. 7E). Comparison of the cell length distributions of epidermal cells revealed that up to ∼190 h the cell populations were similar in the wild type and the mutant, but after 190 h epidermal cells in *er* began to elongate while in the wild type they did not (Fig. 4A and B). This early onset of pavement cell elongation in *er* coincided with early differentiation of meristemoids, GMCs and stomata (Fig. 2A and B). In the wild type, the first asymmetric cell divisions occurred at ∼185 h, the first guard mother cells appeared at ∼200 h, and the first stomata differentiated at ∼240 h; in *er* those events occurred at ∼150 h, ∼170 h, and ∼205 h, respectively. Therefore, the stomata differentiation pathway was initiated ∼35 h earlier in *er* compared to the wild type. By the end of Stage I, a longitudinal row of epidermis in *er* consisted of ∼25 cells and in the cortex of ∼35 cells (Table S1). Therefore, for every epidermal cell there were ∼1.4 cortex cells. This ratio is slightly higher than in the wild type, where for every epidermal cell there were only 1.1 cortex cells (Table S1), and this is presumably due to premature differentiation in the epidermis.

These data suggest that during early pedicel growth, ER directly or indirectly promotes cell elongation in the epidermis and cortex. As cell divisions appear to be dependent on the cell size, the decrease in cell expansion in *er* leads to a significant increase of the cell cycle duration. In addition, the *ER* gene delays cell differentiation in the epidermis, inhibiting formation of stomata lineage cells in one subset of protodermal cells and preventing the transition from symmetric cell divisions to cell elongation in the other.

#### Stage II

During this stage (from 240–340 h), *er* pedicels continued to grow more slowly than the wild type (Fig. 1A). Mutant pedicels increased in length an average of 2.6 times during this stage, while wild type pedicels grew an average of 3.5 times. In the beginning of the stage the average length of *er* cortex cells was similar to the wild type, suggesting that these cells still divided at a standard length (Fig. 6B) but the shift to a smaller cortex cell length evident during this stage in the wild type was absent in *er* (Fig. 7A, B, and F; Table S1). The absence of this shift to smaller cell lengths is made most clear by comparing the average length of the longest 10% of cells (Figure S5; notice the absence of a dip around 340 h in *er*). Analysis of epidermis cell differentiation suggests that while stomata form earlier, they form at a slower rate (Fig. 2C). We did not observe a significant difference in the rate of meristemoid formation, but differentiation of meristemoids into GMC and GMC into stomata was slightly slowed. Since the ER family receptors compete for other components of their signaling pathway [25], in the *er* mutant the ERL1 and ERL2 receptors are expected to have higher activity. The observed decrease in the rate of GMC and stomata differentiation in the *er* mutant is consistent with the proposed function of these receptors in promotion of cell division of meristemoids and inhibition of their differentiation to GMCs. The average epidermal cell length in Stage II did not show a difference between the wild type and *er* (Fig. 3A), except for a consistently larger standard deviation in *er*, indicating a broader cell length distribution than the wild type. Accordingly, we observed that the average length of the shortest 10% of cells was smaller in *er* and the average length of the longest 10% of cells was longer in *er* (Fig. 3C and D). Comparison of *er* epidermal cell length distributions with the wild type demonstrated that the distribution in *er* was broader and flatter between 240–300 h (Fig. 4A and B) but between 300 and 340 h the *er* epidermis had a much larger population of small cells compared to the wild type (Fig. 4C and D). We propose that the increased length of a fraction of the epidermal cells in *er* is due to their early transition to elongation. The presence of a high percentage of smaller cells in the *er* epidermis is likely to be related to multiple factors: decreased overall growth rate of pedicels, increased number of asymmetric cell divisions, and decreased rate of meristemoid and GMC differentiation.

In summary, during Stage II we observed the following changes in the epidermis and cortex of *er*: slower differentiation of stomata, an increased proportion of epidermal cells dividing asymmetrically, a broader range of epidermal cell length, a slower cortex cell growth that is linked with the extended duration of the cell cycle, and no acceleration of the cortex cell cycle towards the end of this stage.

#### Stage III

This stage begins at 340 h, immediately after fertilization, when in the wild type stomata differentiation is completed and both the epidermis and cortex grow by cell elongation. Wild type pedicels are ∼3.05 mm at the beginning of Stage III and increase in length 2.48 times. Pedicels in *er* are ∼1.22 mm long and increase in length 2.29 times (Fig. 1A). Just as in the wild type, the number of stomata in the *er* epidermis reached a maximum at around 340 h (Fig. 2B), indicating the termination of stomata differentiation and the transition of cortex cells to cell expansion. While differentiation of stomata was completed, there were still many meristemoids and GMCs remaining in the epidermis. During Stage III these meristemoids elongated, and a majority of the GMCs remained undifferentiated (Fig. 2B). Pavement cells continue to elongate, although at a slower rate (when measured in the middle of the pedicel) compared to the wild type (Fig. 3B, Fig. 4C, D). The inequality in total pedicel elongation vs. pavement cell elongation is likely related to nonuniform elongation of cells along the proximodistal axis, resulting in the average cell length in the middle of the pedicel not being representative of the overall average cell length. The elongation of cortex cells is very similar in the wild type and in *er*, except they start their elongation at a slightly larger size (Fig. 6B) and at maturity we observe a slight shift to longer lengths in the *er* cell length distribution (Fig. 7G).

In general, we found that the *er* mutation had little impact on cell behavior during this stage, and the short length of pedicels in the mutant can be explained almost entirely by deficiencies acquired during Stages I and II. This indicates that ER does not regulate the extent or rate of cell elongation during Stage III in either the epidermis or the cortex. Since ER does not have an impact on timing of transition to stage III it also does not affect the proliferative window in the epidermis and cortex.

### Modeling cell behavior during pedicel development

The cell length distribution data (Fig. 4 and 7) contains a wealth of information regarding the dynamics of cell proliferation and growth. The overall shape of the cell size distribution is determined by a small number of parameters: the size at which cells divide, the asymmetry (or lack of) in their division, and the rate at which cells grow. Differences in the cell length distribution during growth reflect differences in these basic parameters, and we were interested to examine a simple phenomenological model of cell proliferation and growth in an effort to glean more information from our data. To that end, we constructed a computer simulation based upon a Monte Carlo approach in which individual cell characteristics were randomly sampled from parameter distributions. We made the following assumptions in our model: that the probability a cell will divide at length *l_d_* can be modeled by a normal distribution; that cells divide purely symmetrically; that cells grow only by extending their length, while width remains constant; that cells grow independently of other cells; and that cells grow at a rate proportional to their length, i.e., the instantaneous growth rate *R* = *kl*, where *k* is a constant. To reflect differences in cell behavior over time, simulated growth was divided into four distinct time periods, corresponding to those given in Figures 4 and [7]: 0–190 h, 190–240 h, 240–300 h, and 300–340 h. We did not stimulate cell behavior in wt epidermis after 240 h and in *er* epidermis after 190 h, as this would require the model to comprehend the differential behavior of meristemoids, GMCs, stomata, regular pavement cells, and stomata lineage pavement cells, which would involve adding significant complexity and several new parameters, and our data did not provide sufficiently detailed information to guide that effort.

**Figure 8:**
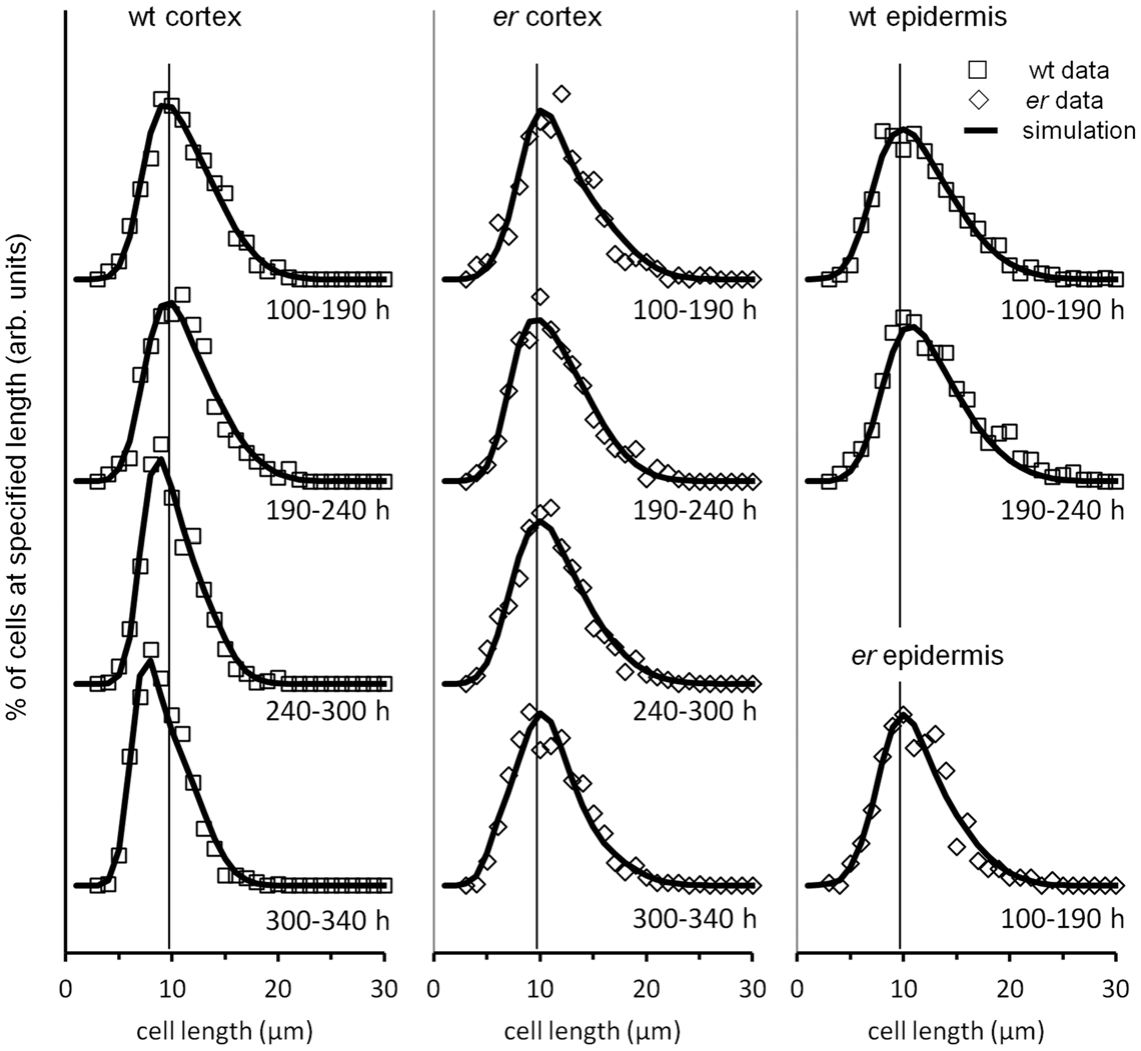
Simulation of *Arabidopsis* pedicel cell size distributions over time. Experimental data (symbols) and simulations (solid lines) of cortex and epidermal cell size distributions. The data for wild type cortex cells is in the left column, for er cortex cells in the middle column, for the wild type epidermal cells is in the top two graphs of right column, and for er epidermal cells is in the lower graph of the right column. Vertical lines at 10 µm are guides to the eye. Later times for epidermis were not simulated.

The results of the simulations and fits are shown in Figure 8, with best-fit cell division length distribution parameters presented in Table 2. While our assumptions were admittedly a simplification of the actual growth process, with them we were able to reproduce the essential features of our data. Good agreement with the data was obtained assuming that the rate constant *k* of cell expansion was constant throughout growth and equaled 0.0132 (h^−1^) for the wild type and 0.0106 (h^−1^) for *er*. Thus our model is consistent with uniform tissue growth being dependent on the constant rate of cell expansion, with individual cells going through independent cell cycle stages. In addition our results demonstrate that the changes in the cell length distributions during pedicel development can be explained solely by changes in the lengths at which cells divide as growth proceeds. As we noticed during analysis of average cell sizes, the sizes at which cortex cells divide in the wild type remained relatively constant until 240 h, when they began to shift to smaller lengths. This shift became more pronounced in the 300–340 h time period but was absent in the *er* mutant (Table 2). In the *er* mutant, cortex cells divide at slightly longer lengths than in the wild type, and the cell size distribution is essentially constant to 300 hours, when it shifts to sizes that are only very slightly shorter.

**Table 2:** Best-fit simulation parameters: mean (μ) and sigma (σ) of the normal distribution specifying the length (mm) at which cells divide.

	wt epidermis		*er* epidermis		wt cortex		*er* cortex	
age (h)	μ	σ	μ	σ	μ	σ	μ	σ
0–190	18.3±0.1	4.0±0.1	17.9±0.2	3.5±0.1	17.3±0.1	3.0±0.1	18.7±0.2	3.5±0.2
190–240	19.3±0.2	3.6±0.1	N/A	N/A	17.4±0.1	3.0±0.1	17.7±0.1	3.3±0.2
240–300	N/A	N/A	N/A	N/A	15.7±0.1	2.0±0.2	17.9±0.1	3.6±0.2
300–330	N/A	N/A	N/A	N/A	14.2±0.2	2.1±0.1	17.1±0.2	3.6±0.2

Uncertainties represent the approximate range over which the parameter gave roughly equivalent goodness-of-fit.

## Discussion

### The model of cell behavior in cortex and epidermis during pedicel development

**Figure 9:**
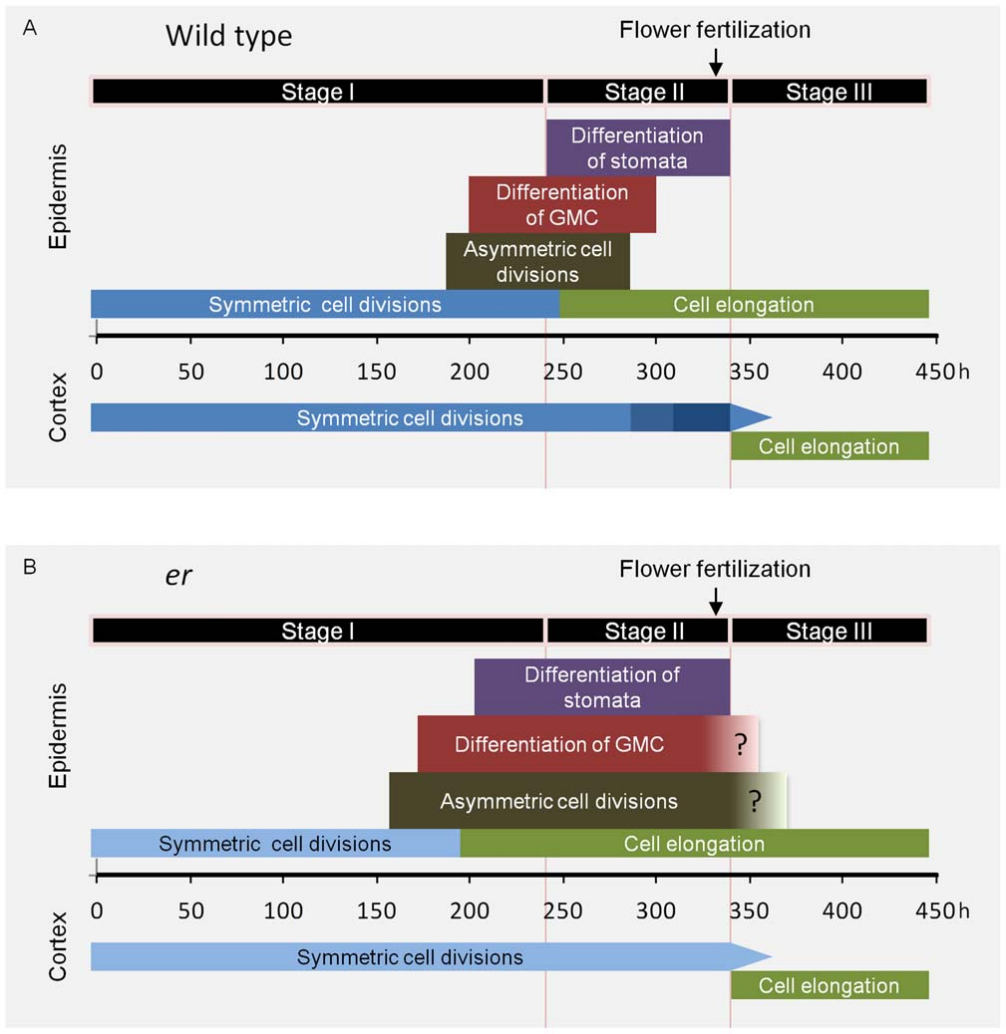
Model of cell behavior in the cortex and epidermis during pedicel growth. The model depicts timing of the events occurring in different layers with the horizontal axis representing time (h). Model for the wild type is in A and for *er* in B. Time zero corresponds to a flower buttress formation on a meristem. The darkness of blue coloring on symmetric cell division tag represents the rate of the cell cycle with darker color representing the faster rate. The narrowing of that tag represents gradual reduction in number of cell divisions. The three stage of pedicel growth are proposed based on observed cell behavior. The timing of all events is based on experimental data except the end of asymmetric cell divisions in the wild type that was inferred and the end of asymmetric cell divisions and GMC differentiation in *er* that are a rough approximation.

To understand mechanisms controlling size and shape of plant organs it is essential to know the contributions of cell proliferation and cell elongation, and how growth is coordinated between different tissue layers. Here we examined pedicel development with the goal to learn more about the cellular basis of growth and pattern formation in plant organs. As a result of our analyses of epidermis and cortex, we propose that there are three stages of pedicel development (Table 1, Fig. 9A).

Stage I is the longest and lasts from pedicel formation to ∼240 hours. At the beginning of this stage both the epidermis and cortex grow solely by symmetric cell divisions. Cells in these two tissue layers are similar in size and divide at the same rate (∼52.5 h). At ∼185 h development in the epidermis begins to change, with some cells dividing symmetrically and some asymmetrically. This change in epidermis development has no impact on the rate of pedicel growth or the behavior of cortex cells. Stage II initiates with the appearance of the first stomata and lasts for ∼100 hours. Interestingly, the onset of stomata differentiation correlates with the termination of symmetric cell divisions in the epidermis, followed closely by the onset of pavement cell elongation. During Stage II the epidermis grows primarily through cell elongation, with proliferation of cells by asymmetric cell division playing a secondary role. The switch to cell elongation requires a transition from the mitotic cell cycle to endoreduplication in epidermal cells that are not part of the stomata differentiation pathway [40]. The timing of that transition, and not differential growth, is the major determinant of ultimate epidermal cell size [41]. At the same time the cell cycle duration in the cortex gradually decreases and cells divide at a smaller size. Whether this is related to the decrease in epidermal cell proliferation and, as a result, the increased availability of a potential cell proliferation factor is an interesting question. By the end of Stage II stomata differentiation is completed. Intriguingly, the end of this stage coincides with differentiation and expansion of cortex cells, and it occurs closely after the appearance of medial vascular bundles [34], the final step of vasculature formation in the pedicel. These observations suggest a temporal coordination of differentiation in different tissue layers during Stage II. Considering that so many changes are occurring in the epidermis and cortex during this stage, it is remarkable that the overall exponential rate of pedicel growth in Stage II is similar to the rate during Stage I. This is somewhat surprising, as the epidermis is believed to control organ growth [42] and one might expect that multiple differentiation events in the epidermis would have an impact on the overall rate of growth. Instead, our observations are consistent with the mechanism determining the rate of organ growth being unaffected by cell differentiation in the epidermis. Stage III is a cell elongation stage and lasts for ∼100 hours. Very little if any cell proliferation occurs during this stage in either the epidermis or the cortex. Overall pedicel growth during this stage transitions from exponential to linear, it is not uniform along the proximodistal axis, and it depends on flower fertilization occurring just before Stage III starts.

It would be most logical to compare the dynamics of pedicel growth to growth of stems, but a detailed temporal analysis of stem growth is lacking. The mechanistic basis of leaf growth, however, has been studied extensively (for a review see [43]). After initiation, two stages of leaf growth are described. Epidermal and mesophyll cells initially proliferate, and then epidermal cells switch to expansion, starting at the distal end of the leaf and progressing towards the proximal end [29]. This transition to cell expansion occurs abruptly in a specified leaf region simultaneously with the differentiation of chloroplasts and onset of photosynthesis, leading to the hypothesis that retrograde transport from chloroplasts regulates the transition to cell expansion in the epidermis [32]. Just as in the cortex of the pedicels, the transition to cell expansion is significantly delayed in the mesophyll compared to the epidermis [29]. Since most chloroplast differentiation and photosynthesis occurs in the mesophyll, it is somewhat surprising that a retrograde signal would induce epidermal but not mesophyll cell expansion. Stomata differentiation precedes pavement cell expansion by approximately one day [32], and continues until the leaf reaches its mature size [44]. For the most part, the leaf growth pattern closely resembles the pattern of pedicel growth. In both cases growth begins with a cell proliferation stage, followed by a period in which stomata differentiate and pavement cells elongate, and ending with a period of mesophyll expansion. In both organs the differentiation of stomata slightly precedes elongation of pavement cells, and the transition to stomata differentiation occurs around day 9–10 ([31], and our data).

Despite the similarities, there are several differences between leaf and pedicel growth. The stomata differentiation period is significantly prolonged in leaves, and the onset of mesophyll expansion does not coincide with the termination of stomata differentiation. The extended stomata formation period is likely necessary for the establishment of a higher stomatal density in leaves compared to pedicels [21]. The transition from cell proliferation to cell expansion follows a longitudinal gradient starting at the tip of the leaf. In pedicels this transition is synchronized along the proximodistal axis. Constant exponential growth depends on uniform behavior of cells. The early termination of exponential growth in leaves (∼day 11; [31]) compared to pedicels (∼day 16) is probably related to the unequal differentiation of leaves along their length. Estimation of the average cell cycle duration in leaf epidermis suggests that it remains constant during development [31], which we also observed in pedicel epidermis during the proliferative phase. However, the cell cycle length in pedicels is significantly longer (∼52.5 h) than in leaves (∼20 h). Comparison of pedicels and the first leaves of *Arabidopsis* illustrates different pathways to reaching similar lengths (∼1 cm first leaves and ∼0.7–0.8 cm pedicels) over similar periods of time (∼20 days; [31]): pedicels grow slowly but have a long period of exponential growth, and leaves at first grow very quickly but terminate their exponential growth early in development.

### The role of ER in regulation of pedicel growth

The *ER* family genes are crucial for the proper growth and differentiation of all aboveground organs [23], [26] however, their precise impact on cell behavior in different tissue layers has not been clear. Our study of pedicel development in *er* demonstrates that the *ER* gene influences the growth rate but not the overall duration of growth. As evidenced from a histological analysis, ER controls the cell cycle duration in the epidermis and cortex. This function is connected to the regulation of cell growth during the proliferative phase as the size at which cells divide is unchanged in the mutant. Multiple factors contribute to efficient cellular growth; the ability to increase the cytoplasmic content, proper expansion of the cell wall, and availability of space to grow [43]. Any of these factors might be regulated by ER. The consistency of cell size at division between the wild type and *er* is an interesting finding, as the existence of a threshold for plant cell division is an undecided issue [7], [31]. Another interesting observation is that the acceleration of the cortex cell cycle, which we observed in the wild type is absent in *er*. If proliferation in the epidermis and cortex is indeed coordinated by a common proliferative factor, the absence of this acceleration could be due to a longer period of asymmetric cell divisions in *er*.

It has been previously reported that in *er* mature epidermis cells are much smaller, and cortex cells are bigger, compared to the wild type [21], [27]. Based on our data, ER is important early on for controlling the rate of cellular growth, but it does not directly regulate the size of mature cells or the rate of cell elongation after cells differentiate. The decreased size of epidermal cells in *er* may be a consequence of the different ratio of epidermal cells to cortex cells and the limited space for epidermal cell elongation. This is consistent with ER expression only in young organ primordia before the cell elongation stage [23], [26]. Recently, the reduced stature of *er* plants was attributed to the function of the gene in the phloem, where it perceives a signal from the endodermis [28]. Some of the vasculature defects caused by *er* mutation are radial expansion of xylem [45] and premature vasculature differentiation [34]. At this point it is difficult to conclusively determine whether *ER* directly regulates growth in the epidermis and cortex during the proliferative stage, or if the reduction of growth in those tissues is a secondary consequence of reduced cellular growth in the vasculature.

The second function of ER is to regulate stomata and pavement cell differentiation in the epidermis (Fig. 9B) by prolonging the proliferative phase and inhibiting premature cell differentiation. We not only observed premature formation of meristemoids in *er*, but also an early onset of pavement cell elongation. The simultaneous shift to early cell differentiation suggests that there is a correlation between onset of the stomata differentiation pathway and the transition to cell elongation in cells that did not become MMCs. While in wild type leaves and pedicels stomata differentiation slightly precedes cell elongation, in the *er* mutant it appears that, at least in pedicel, cell elongation happens first. Therefore, the differentiation of stomata is not a requirement for induction of pavement cell elongation. We hypothesize that cell differentiation in the epidermis is a complex and synchronized event, with some cells differentiating in the stomata pathway and others exiting the mitotic cell cycle and transitioning to expansion. In this way, once the epidermis switches to the differentiation phase, cells have a very limited ability to proliferate unless they are part of the stomata differentiation pathway. More precise temporal analysis of the correlation between cell elongation and stomata differentiation in the wild type and stomata differentiation mutants is needed to verify this hypothesis.

The enhancement of cellular growth by ER during the proliferative stage is limited specifically to the proximodistal axis. A model describing the generation of leaf shape proposes the existence of a PGRAD factor that promotes growth along the proximodistal axis and determines a growth rate constant k [46]. The expression of this factor declines from the proximal to distal ends of the leaf and the factor acts locally. Together with a hypothetical LAM factor it is a major determinant of leaf shape. ER is expressed in a gradient in a leaf [26], promotes the growth rate along the proximodistal axis and is essential for differential growth along this axis in the leaf [27], which makes it a good candidate for being PGRAD. Involvement of ER in regulation of stomata development is consistent with its role in regulation of organ size, as early transition to stomata differentiation should limit the pool of epidermal cells. A study of natural variation in leaf and cotyledon size in wild accessions of *Arabidopsis thaliana* exposed a positive correlation between pavement cell number and size, and a negative correlation with stomata density, suggesting a genetic link between plant organ size and stomata development [47]. Analysis of *ER* signaling pathways in plants with different architectures might help to elucidate whether this family is responsible for different plant morphologies. Unfortunately, the expression of *ER* is tightly regulated at the post-transcriptional level [25], [48] and such analysis is not straightforward.

### Pedicels as a model system for the study of organ growth

Our examination of cell behavior in the epidermis and cortex demonstrates that organ development is an intricate process involving many different events, even without allowing for the high complexity of vasculature differentiation and growth. This elaborateness of cell behavior is likely to be controlled by multiple regulatory pathways functioning at each stage of organ growth. For example, different factors might coordinate and guide organ growth during the proliferative stage versus the cell elongation stage. The contribution of a specific regulatory factor to the control of organ size, the nature of communication between distinct cell layers during growth, and the phenomenon of compensation are intimately connected with the stage of organ development and can only be understood in the temporal context.

Much past research in plant organ growth has focused on the epidermis, as one can observe its development in real time (for review see [49]). However, it is clear that investigation of other tissue behavior is also vital, as those tissues might play critical roles in regulation of plant organ size [28]. Modification of epidermis growth might be a secondary effect caused by changes in internal layers. The present study suggests that pedicels offer a convenient model organ to study the mechanisms regulating plant growth over time and in multiple tissues. Pedicel growth is likely to be a close approximation of stem development, and comparison with leaf growth allows us to identify which mechanisms are common and which are specific to a particular organ. This model system should be useful for examining the impact of other regulatory molecules and environmental factors on plant organ growth.

## Supporting Information

Figure S1 **Pedicels in** ***Arabidopsis*** **.** (A) A pedicel is a structure connecting a flower to a stem. (B) At maturity a pedicel is significantly shorter and broader in *er* compared to the wild type.
(PDF)

Figure S2 An identity of a meristemoid (A), a GMC (B), and a stoma (C) can be determined in the epidermis of pedicels based on their shape.
(PDF)

Figure S3 **Individual pedicel growth plotted over time.** The time is from the moment of the first measurement and does not reflect pedicel age. Pedicels #1, #2 and #3 have reached the expected final size. Pedicels #4 and #5 are shorter than plants that have not been subjected to the stress of repeated handling. Pedicels that have not reached the expected final size were not used for determination of pedicel growth curve in Figure 1A and 8A.
(PDF)

Figure S4 **Image of epidermis at the beginning of Stage II in the wild type.** Pedicel length equaled 1.04 mm, the calculated age was 258 h. At this time GMCs (arrowhead) begin to differentiate into guard cells, pavement cells begin to elongate, and very few divisions occur parallel to proximodistal axis (arrow).
(PDF)

Figure S5 The average size of 10% of the shortest and 10% of the longest cortex cells in the wild type (A) and in *er* (B) over time. Every data point represents an average cell size in an individual pedicel. N = 35–125. Error bars are added to all data points and they are ± SD. The pedicel age (h) was determined based on pedicel length as described in the text.
(PDF)

Table S1 The approximate number of cells in the longitudinal row of epidermis and cortex in the wild type and *er* at an indicated pedicel age (h) as estimated based in pedicel length and the average cell size. For epidermis number of cells corresponds to pavement cells only.
(PDF)

Table S2 Average length (mm) of epidermal and cortex cells at different time periods of pedicel growth in the wild type and *er*. Uncertainties are ± SE. The calculations of average epidermal cell length do not take into account length of meristemoids, guard mother cells and stomata.
(PDF)
