# Supplementary material for: Patterns of Cell Division, Cell Differentiation and Cell Elongation in Epidermis and Cortex of Arabidopsis pedicels in the Wild Type and in erecta
Source: PLoS One. 2012 Sep 25;7(9):e46262. doi: 10.1371/journal.pone.0046262 (PMC3457992; doi:10.1371/journal.pone.0046262)
Supplement: Figure S1 — Pedicels in Arabidopsis . (A) A pedicel is a structure connecting a flower to a stem. (B) At maturity a pedicel is significantly shorter and broader in er compared to the wild type. (PDF) [file pone.0046262.s001.pdf]

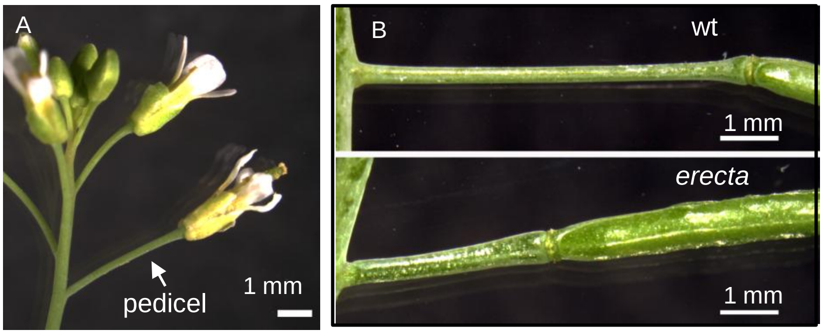

Figure S1. Pedicels in *Arabidopsis*

A. A pedicel is a structure connecting a flower to a stem.

B. At maturity a pedicel is significantly shorter and broader in *er* compared to the wild type.
