# Supplementary material for: Patterns of Cell Division, Cell Differentiation and Cell Elongation in Epidermis and Cortex of Arabidopsis pedicels in the Wild Type and in erecta
Source: PLoS One. 2012 Sep 25;7(9):e46262. doi: 10.1371/journal.pone.0046262 (PMC3457992; doi:10.1371/journal.pone.0046262)
Supplement: Figure S2 — An identity of a meristemoid (A), a GMC (B), and a stoma (C) can be determined in the epidermis of pedicels based on their shape. (PDF) [file pone.0046262.s002.pdf]

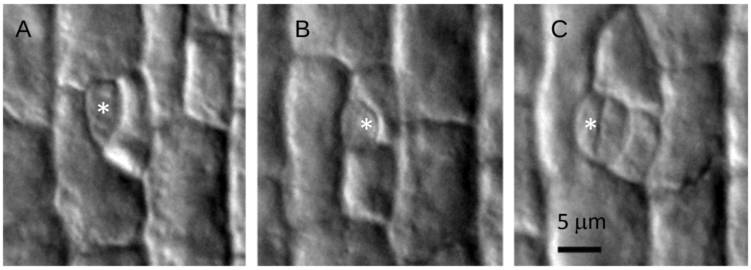

Figure S2. An identity of a meristemoid (A), a GMC (B), and a stoma (C) can be determined in the epidermis of pedicels based on their shape.
