# Supplementary material for: Patterns of Cell Division, Cell Differentiation and Cell Elongation in Epidermis and Cortex of Arabidopsis pedicels in the Wild Type and in erecta
Source: PLoS One. 2012 Sep 25;7(9):e46262. doi: 10.1371/journal.pone.0046262 (PMC3457992; doi:10.1371/journal.pone.0046262)
Supplement: Figure S3 — Individual pedicel growth plotted over time. The time is from the moment of the first measurement and does not reflect pedicel age. Pedicels #1, #2 and #3 have reached the expected final size. Pedicels #4 and #5 are shorter than plants that have not been subjected to the stress of repeated handling. Pedicels that have not reached the expected final size were not used for determination of pedicel growth curve in Figure 1A and 8A. (PDF) [file pone.0046262.s003.pdf]

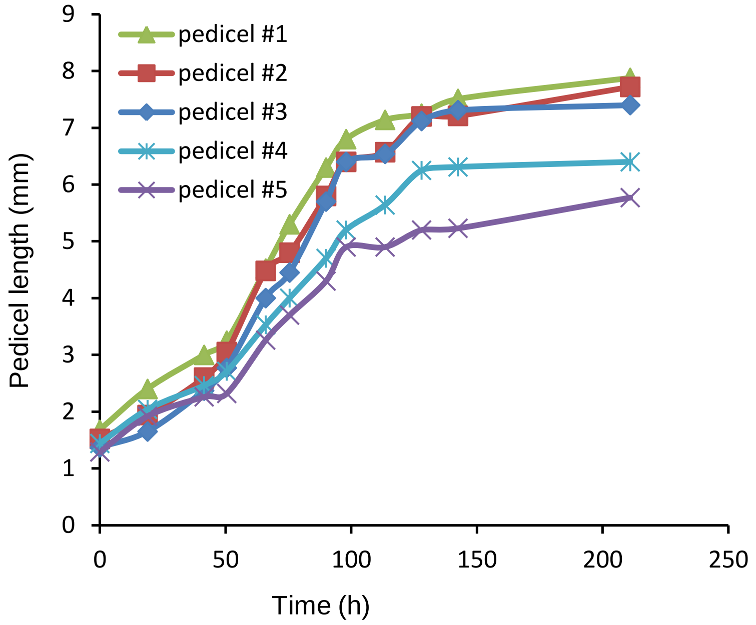

Figure S3 . Individual pedicel growth plotted over time.
The time is from the moment of the first measurement and does not reflect pedicel age. Pedicels #1, #2 and #3 have reached the expected final size. Pedicels #4 and #5 are shorter than plants that have not been subjected to the stress of repeated handling. Pedicels that have not reached the expected final size were not used for determination of pedicel growth curve in Figure 1A and 8A.
