# Supplementary material for: Patterns of Cell Division, Cell Differentiation and Cell Elongation in Epidermis and Cortex of Arabidopsis pedicels in the Wild Type and in erecta
Source: PLoS One. 2012 Sep 25;7(9):e46262. doi: 10.1371/journal.pone.0046262 (PMC3457992; doi:10.1371/journal.pone.0046262)
Supplement: Figure S4 — Image of epidermis at the beginning of Stage II in the wild type. Pedicel length equaled 1.04 mm, the calculated age was 258 h. At this time GMCs (arrowhead) begin to differentiate into guard cells, pavement cells begin to elongate, and very few divisions occur parallel to proximodistal axis (arrow). (PDF) [file pone.0046262.s004.pdf]

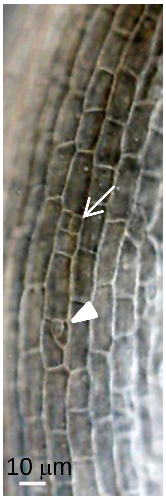

Figure S4. Image of epidermis at the beginning of Stage II in the wild type.
Pedicel length equaled 1.04 mm, the calculated age was 258 h. At this time GMCs (arrowhead) begin to differentiate into guard cells, pavement cells begin to elongate, and very few divisions occur parallel to proximodistal axis (arrow).
