# Supplementary material for: Patterns of Cell Division, Cell Differentiation and Cell Elongation in Epidermis and Cortex of Arabidopsis pedicels in the Wild Type and in erecta
Source: PLoS One. 2012 Sep 25;7(9):e46262. doi: 10.1371/journal.pone.0046262 (PMC3457992; doi:10.1371/journal.pone.0046262)
Supplement: Figure S5 — The average size of 10% of the shortest and 10% of the longest cortex cells in the wild type (A) and in er (B) over time. Every data point represents an average cell size in an individual pedicel. N = 35–125. Error bars are added to all data points and they are ± SD. The pedicel age (h) was determined based on pedicel length as described in the text. (PDF) [file pone.0046262.s005.pdf]

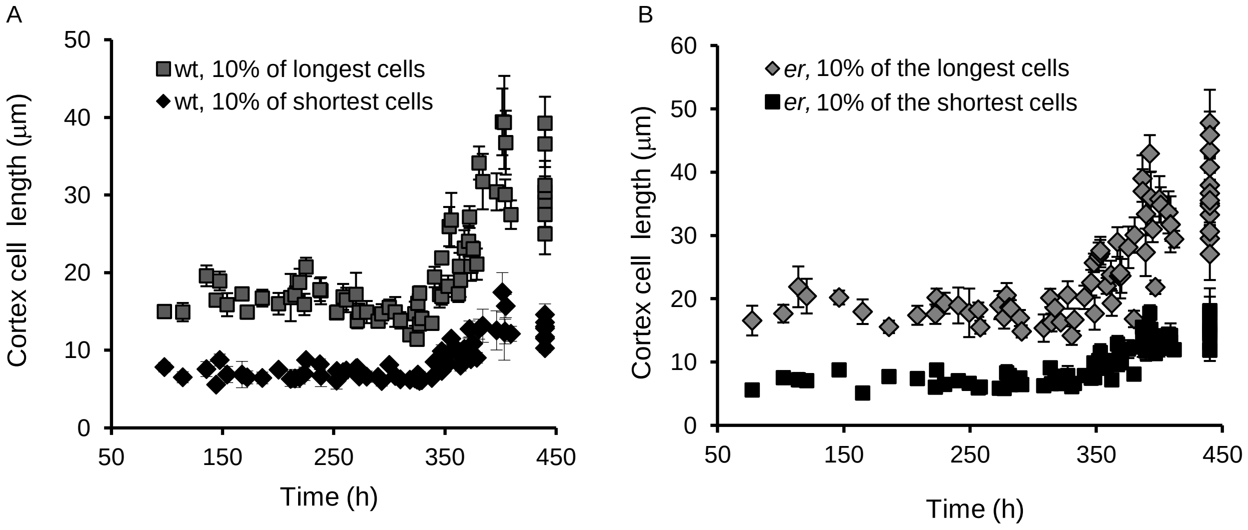

Figure S5. The average size of 10% of the shortest and 10% of the longest cortex cells in the wild type (A) and in *er* (B) over time. Every data point represents an average cell size in an individual pedicel. N=35-125. Error bars are added to all data points and they are ± SD. The pedicel age (h) was determined based on pedicel length as described in the text.
