# Supplementary material for: Patterns of Cell Division, Cell Differentiation and Cell Elongation in Epidermis and Cortex of Arabidopsis pedicels in the Wild Type and in erecta
Source: PLoS One. 2012 Sep 25;7(9):e46262. doi: 10.1371/journal.pone.0046262 (PMC3457992; doi:10.1371/journal.pone.0046262)
Supplement: Table S1 — The approximate number of cells in the longitudinal row of epidermis and cortex in the wild type and er at an indicated pedicel age (h) as estimated based in pedicel length and the average cell size. For epidermis number of cells corresponds to pavement cells only. (PDF) [file pone.0046262.s006.pdf]

Table S1. The approximate number of cells in the longitudinal row of epidermis and cortex in the wild type and *er* at an indicated pedicel age (h) as estimated based in pedicel length and the average cell size. For epidermis number of cells corresponds to pavement cells only.

	wt	*er*	wt	*er*
age (h)	epidermis	epidermis	cortex	cortex
190	34	21	36	20
240	67	25	74	35
300	75	30	180	69
340	74	33	320	90
