# Supplementary material for: Patterns of Cell Division, Cell Differentiation and Cell Elongation in Epidermis and Cortex of Arabidopsis pedicels in the Wild Type and in erecta
Source: PLoS One. 2012 Sep 25;7(9):e46262. doi: 10.1371/journal.pone.0046262 (PMC3457992; doi:10.1371/journal.pone.0046262)
Supplement: Table S2 — Average length (mm) of epidermal and cortex cells at different time periods of pedicel growth in the wild type and er. Uncertainties are ± SE. The calculations of average epidermal cell length do not take into account length of meristemoids, guard mother cells and stomata. (PDF) [file pone.0046262.s007.pdf]

Table S2. Average length (mm) of epidermal and cortex cells at different time periods of pedicel growth in the wild type and *er*. Uncertainties are ± SE. The calculations of average epidermal cell length do not take into account length of meristemoids, guard mother cells and stomata.

	wt	*er*	wt	*er*
age (h)	epidermis	epidermis	cortex	cortex
100-190	12.2±0.5	11.9 ±0.2	11.4 ±0.4	12.2 ±0.4
190-240	13.2±0.4	15.1 ±0.9	11.7 ±0.4	11.9 ±0.4
240-300	18.4±0.8	21.1 ±0.8	10.6 ±0.2	11.4 ±0.3
300-340	35.4±1.7	31.1±2.9	9.7±0.2	11.7 ±0.5
340-400	59.8±4.2	51.8 ±2.1	15.9 ±0.8	18.6 ±0.8
>440	94.3±4.2	60.5±2.7	20.7 ±1.2	24.8 ±1.1
